# Behavioral Immunity Suppresses an Epizootic in Caribbean Spiny Lobsters

**DOI:** 10.1371/journal.pone.0126374

**Published:** 2015-06-10

**Authors:** Mark J. Butler, Donald C. Behringer, Thomas W. Dolan, Jessica Moss, Jeffrey D. Shields

**Affiliations:** 1 Department of Biological Sciences, Old Dominion University, Norfolk, VA, 23529, United States of America; 2 School of Forest Resources and Conservation, Program in Fisheries and Aquatic Sciences and Emerging Pathogens Institute, University of Florida, Gainesville, FL, 32653, United States of America; 3 Bureau of Fisheries, USVI Department of Planning & Natural Resources, Frederiksted, VI 00840, USVI; 4 Virginia Institute of Marine Science, The College of William & Mary, Gloucester Point, Virginia, 23062, United States of America; Linneaus University, SWEDEN

## Abstract

Sociality has evolved in a wide range of animal taxa but infectious diseases spread rapidly in populations of aggregated individuals, potentially negating the advantages of their social interactions. To disengage from the coevolutionary struggle with pathogens, some hosts have evolved various forms of “behavioral immunity”; yet, the effectiveness of such behaviors in controlling epizootics in the wild is untested. Here we show how one form of behavioral immunity (i.e., the aversion of diseased conspecifics) practiced by Caribbean spiny lobsters (*Panulirus argus*) when subject to the socially transmitted PaV1 virus, appears to have prevented an epizootic over a large seascape. We capitalized on a "natural experiment" in which a die-off of sponges in the Florida Keys (USA) resulted in a loss of shelters for juvenile lobsters over a ~2500km^2^ region. Lobsters were thus concentrated in the few remaining shelters, presumably increasing their exposure to the contagious virus. Despite this spatial reorganization of the population, viral prevalence in lobsters remained unchanged after the sponge die-off and for years thereafter. A field experiment in which we introduced either a healthy or PaV1-infected lobster into lobster aggregations in natural dens confirmed that spiny lobsters practice behavioral immunity. Healthy lobsters vacated dens occupied by PaV1-infected lobsters despite the scarcity of alternative shelters and the higher risk of predation they faced when searching for a new den. Simulations from a spatially-explicit, individual-based model confirmed our empirical results, demonstrating the efficacy of behavioral immunity in preventing epizootics in this system.

## Introduction

Sociality confers certain advantages to animals and has nurtured the evolution of complex social systems, but it has evolved with costs [1–3]. Socially transmissible pathogens often spread more efficiently and evolve greater virulence within patches of aggregated hosts, diminishing the benefits of host social behavior [4,5]. The importance of local or "household" interactions in the transmission of communicable diseases among hosts [6–8] is highlighted by how effective the segregation of hosts can be in reducing the spread of pathogens [9–11]. Although a host’s immune system functions as the primary defense against pathogens once acquired, behaviors that isolate infected conspecifics can serve as a first line of defense that reduces encounters with pathogens [12,13]. For example, humans display “behavioral immunity” when infected individuals are socially ostracized or quarantined [14]. Indeed, human xenophobic behavior and “disgust” toward out-groups may have evolved as cultural traits that reduce the transmission of pathogens [14,15]. Behavioral strategies that curtail the spread of parasites and pathogens have also evolved in wild animal populations.

Some of the most sophisticated behaviors that serve to minimize the spread of pathogens and parasites evolved in eusocial animals. For example, some species of ants and bees when infected exhibit "altruistic suicide" by abandoning their colonies to prevent further transmission [16–18]. Others engage in sophisticated behaviors and use of poisons to disinfect their colonies and prevent epizootics [19]. The converse of such altruistic behaviors occurs when uninfected members of the population drive away or avoid infected conspecifics [20, 21], not unlike quarantine strategies used by humans. A dramatic example of this occurs in wild populations of the Caribbean spiny lobster (*Panulirus argus*), a social species that shares and defends dens with conspecifics. However, healthy spiny lobsters are repelled by odors produced by diseased lobsters [22,23] and avoid sharing dens with lobsters infected with a pathogenic virus (*Panulirus argus* virus 1; PaV1) [24].

### The Caribbean Spiny Lobster—PaV1 virus Pathosystem

Spiny lobsters are an iconic and abundant species in the Caribbean where they support one of the most valuable commercial and recreational fisheries in the region, whose estimated value exceeds $500M US annually [25]. Spiny lobsters have a complex life history and, unlike clawed lobsters, are social and generally non-aggressive during most of their juvenile and adult stages. Their life history begins with the spawning of larvae from adult females that dwell on coral reefs. Larvae spend a 6–8 mos in the oceanic plankton before returning to coastal nursery areas during their final postlarval stage. After spending another 1–3 mos hidden within clumps of macroalgae or other vegetation, juvenile lobsters then emerge from their vegetated settlement habitat to forage at night and seek shelter by day in crevice dens (e.g., large sponges, coral heads, and rocky crevices) that they share with conspecifics. From this point on in their life cycle, Caribbean spiny lobsters are social and use chemical cues to locate and dwell with conspecifics for the remainder of their life.

PaV1 (Panulirus argus virus 1) is an undefined icosahedral dsDNA virus first described in 2000 from infected lobsters in the Florida Keys, Florida (USA) [24]. Since then it has been reported in spiny lobsters throughout much of the Caribbean where its prevalence ranges from 0–17% [26]. Postlarval and juvenile spiny lobsters are highly susceptible to infection by the PaV1 virus, but susceptibility decreases with increasing lobster size [27]. Once infected, juveniles typically die within 2–8 weeks. Conversely, adult lobsters are potential carriers of the virus, as they can be infected but remain asymptomatic [27]. Transmission of PaV1 occurs readily among juvenile lobsters through close contact and over short distances in water, but waterborne transmission has not been reported for the larger juveniles relevant to this study [27]. Of particular importance to the issue of behavioral immunity is our finding that healthy juvenile lobsters use chemical cues to avoid infected lobsters and can detect diseased lobsters before they become infectious [22,23]. The avoidance of PaV1-infected lobsters by healthy lobsters relegates infected lobsters to dens by themselves, which presumably limits PaV1 transmission. However, this hypothesis was untested until the widespread and rapid loss of sponges used as dens by juvenile lobsters in the Florida Keys (Florida, USA) afforded the opportunity to test the effectiveness of lobster behavioral immunity over a large, natural seascape.

**Fig 1:**
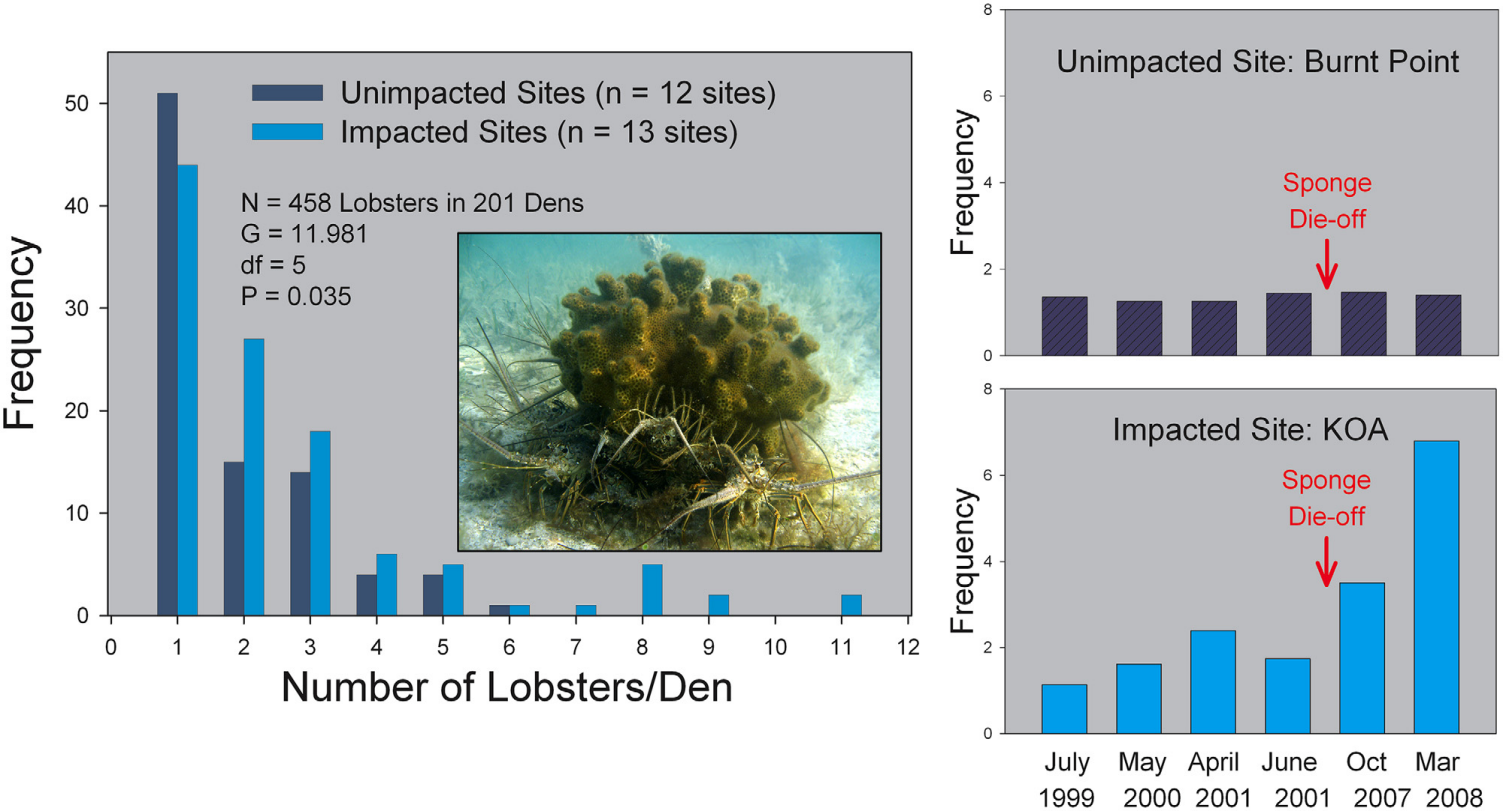
Lobster den aggregation patterns at sites impacted by the sponge die-off versus those at unimpacted sites. (Left) Frequency distribution showing the number of lobsters observed per den in October 2007 for over 200 dens and over 450 lobsters surveyed on sites that were unimpacted (dark blue; N = 12 sites) or impacted (light blue; N = 13 sites) by the sponge die-off. Note the greater frequency of dens observed with two or more lobsters per den on impacted sites. (Inset) Photo of an aggregation of healthy lobsters in a coral den following the 2007 sponge die-off. (Right) Examples of time-series of lobster den aggregation patterns at two sites before and after the sponge die-off: one site unimpacted by the sponge die-off (top) and one impacted (bottom) by the sponge die-off. Note the increase in lobster aggregation size after the sponge die-off at the impacted site.

In August-September 2007, nearly the entire sponge community in a large area (~2500 km^2^) of the Florida Keys was decimated by the occurrence of a persistent bloom of cyanobacteria (*Synechococcus* spp.), similar to circumstances observed two decades earlier [28]. Large sponges (> 20 cm dia) that provide the majority of shelters to juvenile lobsters, and whose density averaged 4700/ha prior to the die-off, were all killed within the area impacted by the cyanobacteria bloom. All that remained as shelter for juvenile lobsters after the die-off were scattered coral heads and rocky crevices representing < 5% of the original den density. Juvenile lobsters are unable to emigrate from large areas of disturbed habitat [29]; thus as sponges disappeared, lobsters shared the few remaining shelters, creating unusually large aggregations on sites subject to the sponge die-off as compared to unimpacted sites (Fig 1). Moreover, the loss of natural dens for juvenile lobsters brought about by the sponge die-off placed healthy lobsters in a dilemma. If a PaV1-infected lobster was present in a den, an uninfected individual could either share the den and risk infection (and eventual death), or search for another den but risk predation while traveling in the open.

Despite the over-aggregation of lobsters in such a large area, we hypothesized that the aversion of healthy lobsters to diseased conspecifics would limit density-dependent pathogen transmission, and thus the prevalence of PaV1 would not increase. To examine this hypothesis, we undertook three lines of investigation. Our objectives were to (1) conduct a field test of competition for shelter between healthy versus diseased lobsters in the affected area, (2) model the effect of this massive social reorganization of lobsters to estimate potential changes in the prevalence of PaV1 infection, and (3) evaluate model predictions with empirical estimates of disease prevalence before and after the sponge die-off. Our findings indicate that behavioral immunity is operating in this natural host-pathogen system and appears to limit the development of epizootic outbreaks.

## Methods

### Ethics Statement

No protected, endangered, or vertebrate species were used in this investigation. Most lobsters used in this study were simply observed; if collected, most were released unharmed after acquisition of a blood sample through non-lethal sampling or after use in the tethering experiment. Only naturally PaV1-infected lobsters collected near the study area were used in field experiments and no lobsters infected with PaV1 were released into the wild. Lobsters collected for histological examination of tissues were anesthetized on ice prior to dissection. This research was conducted under permits granted by the Everglades National Park (EVER-2006-SCI-0031, EVER-2009-Sci-0060, EVER-2012-SCI-0034) and Florida Fish and Wildlife Conservation Commission (SAL-06SRR-582).

### Field Test of Behavioral Immunity in Lobsters

We tested whether aggregations of healthy lobsters in natural dens differed in their response to the introduction into their den of an additional healthy lobster or a PaV1-infected lobster in a field experiment (locations: 24 56 29.72°N, 80 49 24.58°W; 24 50 30.55°N, 80 47 44.86°W; 24 50 17.04°N, 80 48 05.18°W) conducted on sites that had experienced the sponge die-off and thus where lobsters were highly aggregated. Lobsters (16–56 mm CL) were first collected by divers from natural habitats in the middle Florida Keys region, but not at the study site. The presence or absence of PaV1 in collected lobsters was assayed using PCR and all diseased lobsters used in the experiment were visibly diseased (i.e., showed clinical signs of infection). Lobsters were tethered by tying a loop of monofilament (8 kg test) around their carapace (between the second and third periopods) with a snap swivel tied on the dorsal side of the carapace and the knot sealed with cyanoacrylate superglue. The snap swivel was clipped to a brick by a 50 cm long monofilament line. This arrangement permitted tethered lobsters to move freely and naturally within the limits of the tether, but they could not escape. Divers introduced either a single healthy or single visibly diseased juvenile lobster into separate, haphazardly selected natural aggregations of lobsters found under coral heads or within rocky holes. Dens were individually marked with numbered surface buoys and their GPS positions recorded so they could be relocated. We counted and estimated the sizes of all lobsters within each den prior to the introduction of the tethered lobster and then again 24 hrs later; the size of the lobster aggregation did not differ significantly between treatments (mean = 3.8 lobsters/den; diseased lobster addition den mean = 4.0 lobsters/den) and lobsters in aggregations were similar in size in both treatments (mean = 32 mm CL healthy lobster addition treatment vs. 37 mm CL disease lobster addition treatment; range: 20–55 mm CL). Healthy tethered lobsters were released after the study, whereas diseased lobsters were returned to the laboratory and euthanized to prevent further spread of the PaV1 virus. Lobsters were tethered in a total of 46 dens (tethered lobsters: 24 healthy, 22 diseased) that initially contained 177 lobsters with aggregations ranging from 2–15 lobsters per den (mean & sd of aggregation size: 4 ± 2). These tethering data were analyzed using a 2-way log-linear contingency table analysis.

### Simulation Modeling of Potential Effectiveness of Behavioral Immunity in the Wild

Earlier versions of our individual-based spatially-explicit model were developed to describe lobster recruitment dynamics in response to habitat and climate changes [29–31]. The model was subsequently modified to include PaV1 disease dynamics and to test the evolution and timing of social aversion as a means of limiting the transmission of PaV1 between spiny lobsters [8]. In this application of the model, we explored the effects of the sponge die-off on PaV1 prevalence in juvenile lobsters given behavioral immunity. A detailed description of the model, including sensitivity analyses of the parameters of the model of disease transmission and avoidance, is published elsewhere [8], so here we briefly describe the model’s general structure and the simulations conducted for this study. The model was coded in C++ using Microsoft Visual Studio 2005.

#### Spatial Structure of the Model

The model’s spatial domain consisted of a spatially-explicit seascape "map" of the primary juvenile lobster habitat in the Florida Keys and consisted of 2792 contiguous 1 km^2^ square habitat cells (seagrass, sand/mud, hard-bottom, or land) that correspond with true habitat locations determined from GIS data. Hard-bottom cells contained realistic densities of several types of benthic structures, including several species of large sponges, whose density and size were previously determined from diver-based field surveys at more than 300 sites. The density of sponges and other lobster shelters at unsampled locations in the model's seascape were generated with ordinary kriging and each shelter was randomly assigned a lobster carrying capacity as determined from the maximum lobster group sizes for each shelter type recorded by divers in repeated surveys.

#### Lobster Life History

**Fig 2:**
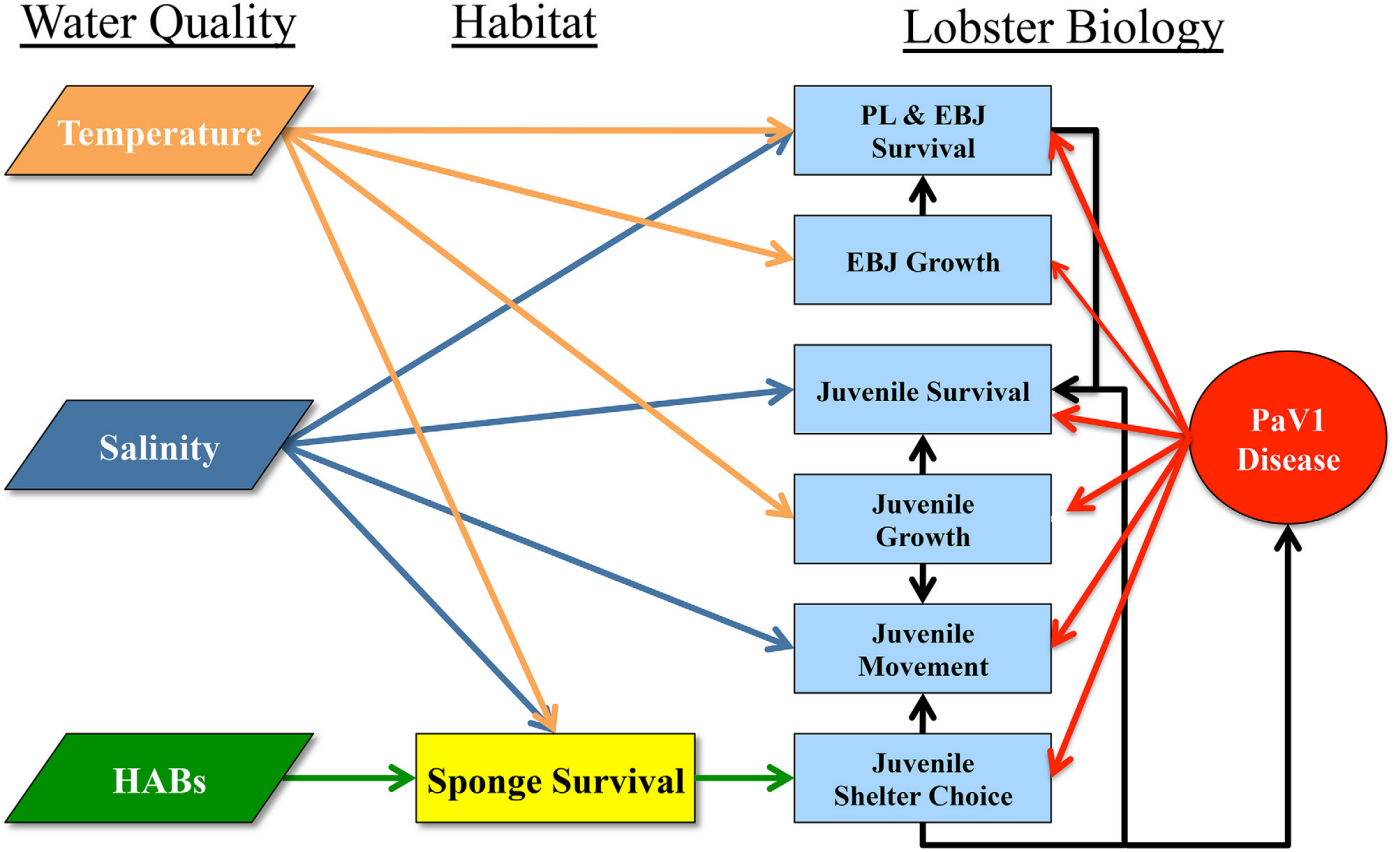
Schematic diagram summarizing the interrelationships among water quality, nursery habitat, and lobster biology that were modeled in our simulation of PaV1 prevalence in lobsters before and after the sponge die-off. Of particular relevance to the simulations are: (1) the effects of harmful algal blooms (HABS) on sponge survival and thus shelter for juvenile lobsters; and (2) the myriad effects of PaV1 infection on lobster biological processes.

The model operated on a daily time step composed of a sequence of processes that mimic the daily dynamics of real lobsters, including the settlement of postlarvae followed by the movement, shelter selection, growth, and mortality of juvenile lobsters (Fig 2). The model focused on juvenile lobster population dynamics until they reached 50 mm CL and thus did not include adult lobster dynamics or interactions with the lobster fishery. Each lobster was individually represented in the model with regard to its location, size, age, den use, and PaV1 infection status.

New lobsters entered the model every 28 days in the 10 yr simulation, which mimics the natural, pulsed lunar arrival of postlarvae to the coastal environment. Monthly variation in the number of postlarvae arriving in the model was based on a 10 yr empirical time-series collected and provided by the Florida Fish and Wildlife Conservation Commission. The number of new postlarval recruits entering the model each month was obtained by scaling the monthly deviation from the ten-year average to an average of 114,500 new individuals/mo in the model. The postlarvae entering the model domain each month were distributed uniformly among cells of similar habitat without regard to their geographic location. Based on experiments involving habitat preferences at settlement [32], we distributed (i.e., "settled") 17% of the new individuals each month in seagrass cells and 83% of the new individuals with macroalgae-dominated hard-bottom cells.

Initial sizes (i.e., carapace lengths) were randomly assigned to individual early benthic stage lobsters in the model based on a truncated normal distribution with a mean of 6.3 mm CL and a standard deviation of 0.3 based on empirical data [33]. Growth of individual lobsters was simulated in discrete steps, reflecting the molting process of arthropods and based on laboratory and field growth experiments [34]. Individuals were evaluated daily to determine whether they molted, which was determined by each individual's size and the number of days since its last molt. If molting occurred, then growth (i.e., length increase) was determined based on the current size of the lobster.

Shelter selection depended on lobster body size, lobster density, and the habitat cell type (i.e., seagrass or hard-bottom cell). The type of shelter utilized affected lobster mortality and the movement of lobsters among cells, which was represented in other subroutines. Shelter selection rules were represented separately for algal-stage juveniles (< 12 mm CL) and postalgal-stage juveniles (25 to 50 mm CL). Transitional juveniles (13 to 25 mm CL) sheltered both in macroalgae, as did algal-stage juveniles, and in crevice shelters, as did postalgal juveniles. Shelter selection rules were formulated based on field data and laboratory investigations of habitat choice [34–36]. All algal-stage juveniles remained in seagrass or macroalgae, as is their nature. However, larger postalgal-stage juveniles left their daytime shelters to forage in surrounding areas each night so chose new shelters each morning based on size-dependent shelter preferences, shelter availability, and the presence of other lobsters—both healthy and infected.

The daily probability of mortality for a lobster was computed as a weighted sum of day, night, and twilight mortality probabilities with each dependent on lobster size and shelter use. Daytime was considered to be 12 hrs, twilight was 2 hrs, and nighttime was 10 hrs. We determined the probability of mortality for each of the daytime, twilight, and nighttime periods based on the shelter inhabited by the lobster for each of the periods, and then computed a total daily probability by weighting the three probabilities by the hours of each period. Mortality probabilities were determined through least-squares fitting of equations to size-specific and shelter-specific mortality data derived from field tethering and mark-recapture studies [37–39].

The probability of movement of lobsters among habitat cells was a function of lobster size and the availability of shelter in their habitat cell. The functions that described the size-specific probability of movement for lobsters in different types of shelter are based on mark-recapture records for over 500 individual lobsters in two dozen 2500 m^2^ field sites within hard-bottom habitat [40]. Small juvenile lobsters that have found shelter rarely move to other shelters and typically forage at night within a meter or two of their daytime shelter [40]. Thus, small individuals < 30 mm CL that occupied a shelter were assumed to remain in their cell. If the movement algorithm indicated that an individual lobster was selected to migrate, then the individual was moved to a randomly selected cell from among the neighboring habitat cells. The model incorporated deflecting boundaries, which allowed lobsters to move along edges but not to leave the model domain.

Detailed descriptions, algorithms, and data sources for the PaV1 disease dynamics in the model are published elsewhere [8], but an overview is given here and depicted in Fig 2. Initial prevalence of PaV1 infections was achieved by randomly infecting individuals in each habitat cell based on the prevalence (mean = 8.5%; sd = 13.3%) observed in 183 lobsters at 14 sites in June-July 2007 prior to the sponge die-off. PaV1 then entered the model domain each month with the arrival of infected postlarvae, the proportion of which was based on empirical estimates of monthly prevalence observed in newly recruited postlarvae [41]. Thereafter, transmission of PaV1 among juveniles was modeled based on den-sharing interactions between uninfected and infected lobsters. Transmission occurred based on individual susceptibility to infection, a biomass-dependent amount of virus to which a susceptible lobster was exposed while sharing a den, and the time course of the disease in any infected lobsters that shared a den with the focal lobster. Laboratory assays indicate that newly infected lobsters move at similar rates as uninfected lobsters, but become sedentary once the disease progresses and lobsters become infectious [42]. Therefore, in the model we stipulated that lobster movement remained unchanged until it became infectious, thereafter its probability of emigration among habitat cells was set to zero and the lobster no longer changed shelter unless forced out by another lobster. Disease also affected the shelter selection routine through competition for dens between healthy and infected lobsters. Uninfected lobsters would not share dens with infected lobsters once they became chemically detectable.

We simulated sponge-die offs in the area of the model corresponding to areas of Florida Bay that experienced the cyanobacteria bloom and resultant sponge die-off in 2007. Sponge populations declined within each model cell based on the number of days of exposure to the harmful algal bloom and species-specific mortality functions. No regrowth of the sponge community was permitted during each simulation as little recovery of large sponges suitable for lobster occupancy could have occurred over that time period.

We ran three simulations. In two simulations we modeled disease dynamics with and without the occurrence of sponge die-offs, and those simulations included the avoidance of PaV1 infected lobsters by uninfected lobsters. A third simulation was run with sponge die-off conditions but we “turned off” behavioral immunity in lobsters so that uninfected lobsters no longer detected and avoided infected lobsters. This third simulation provided a hypothetical baseline against which we could examine the effectiveness of behavioral immunity in constraining the spread of the PaV1 virus. Each model simulation ran for 10 yrs and 10 replicates per simulation were run, which exceeded the minimum sample size necessary to detect a 1% difference from the mean prevalence at the α = 0.05 level as estimated from a Visual Jackknife technique. Each simulation replicate tracked as many as 750,000 individual lobsters in the model at one time and generated tens of billions of unique individuals over each 10 yr simulation. The potential output of individual-based simulations like these is nearly limitless, but we focused on two metrics: (a) the abundance of juvenile lobsters in the 45–50 mm CL size range (a size that represents the lobsters that survived the cumulative effects of PaV1-induced juvenile mortality) and (b) the prevalence of PaV1 in this same group of juvenile lobsters.

### Empirical Time-Series of PaV1 Prevalence in Lobsters

The “acid-test” of our simulation model predictions came from our field studies of PaV1 prevalence before and after the sponge die-off. Since 2000, we had been monitoring PaV1 infection in juvenile lobster populations at 10 nursery sites in the same region of the Florida Keys as depicted in our simulation model. This time-series of data allowed us to evaluate the prevalence of PaV1 in lobsters just before the sponge die-off (May-June 2007), eight months after the sponge die-off (May-June 2008), and then annually for five years after the sponge die-off (2009–2013). During each 30-min survey, two divers collected every juvenile lobster that they encountered on each site, and each lobster was then assessed for PaV1 infection by one or more of three commonly used methods: visual inspection for clinical signs of disease [24], histological assessment of tissue pathology (foregut, hindgut, heart, hepatopancreas, gill, tail muscle) in prepared tissues [24], and PCR analysis of hemolymph (blood) samples [41,43]. PCR is by far the most accurate of the three methods in detecting the presence of the PaV1 virus, but earlier studies have used all three methods [44] so we present each of them for comparative purposes.

## Results

### Field Test of Behavioral Immunity in Lobsters

**Fig 3:**
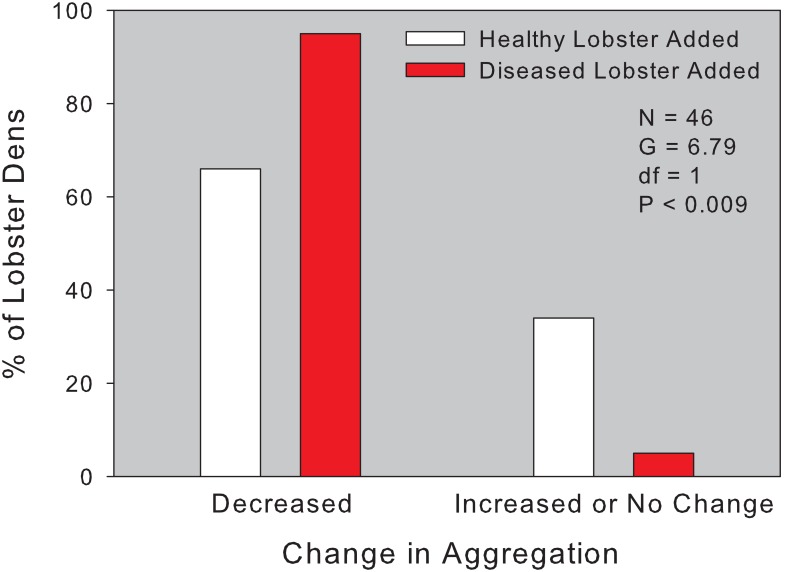
Results of a field experiment testing the response of lobsters aggregated in dens to the introduction of a healthy or clinically infected lobster. Histograms show changes after 24 hrs in the occupancy of dens by wild lobsters after a tethered lobster (diseased or healthy) was introduced into each den. Bars represent the proportion of the 46 dens (containing a total of 177 lobsters) in which lobster abundance decreased (i.e., lobsters left the den) vs. increased/unchanged (i.e., lobsters remained or new ones entered the den). Statistical results of a log-linear contingency table analysis are also shown.

The normal nightly reshuffling of lobsters among dens, along with emigration from dens that might be caused by diver disturbance, resulted in the departure of some lobsters from nearly all dens. However, significantly more lobsters vacated dens when a PaV1-infected lobster was added to their aggregation as compared to the addition of an uninfected lobster (n = 46; G = 6.79; df = 1; P < 0.009; Fig 3). This is consistent with laboratory studies of den competition [15] and confirms the powerful influence of behavioral immunity on lobster den choice. Yet, it remained to be demonstrated whether this behavioral immunity was effective in preventing the spread of the pathogen after the sponge die-off forced lobsters into unnaturally large aggregations.

### Simulation Modeling of Potential Effectiveness of Behavioral Immunity in the Wild

**Fig 4:**
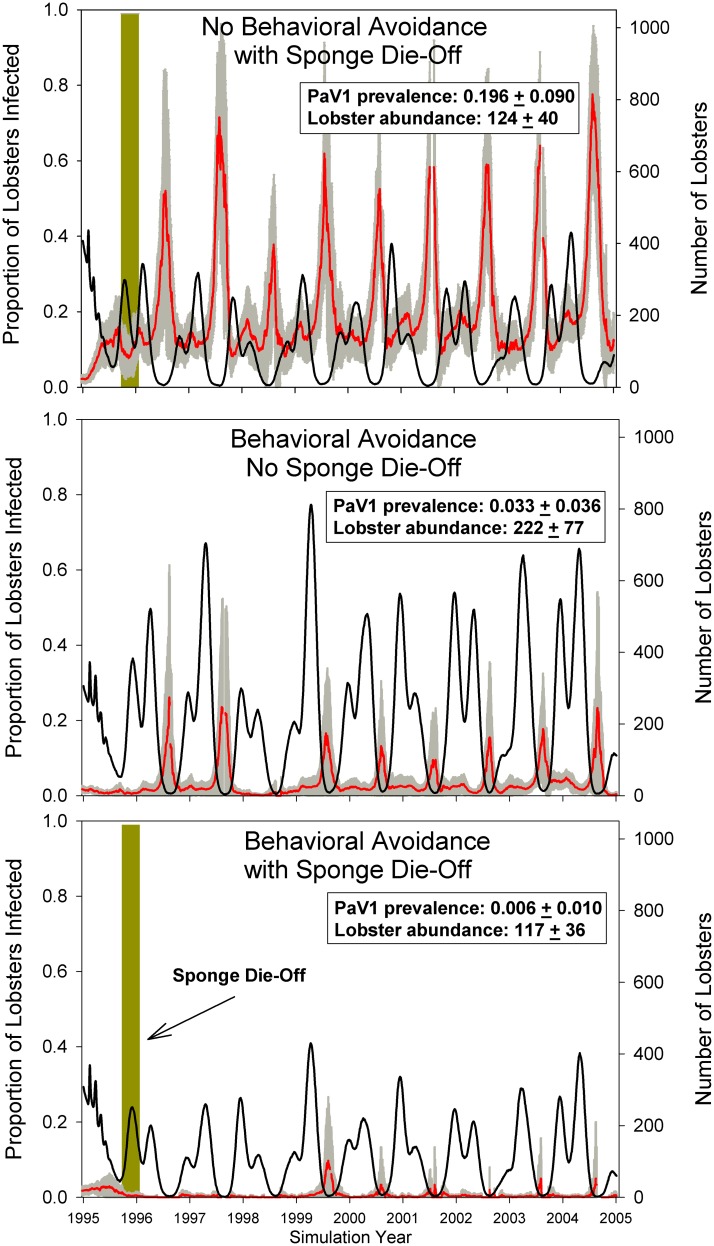
Computer simulations demonstrating the effectiveness of behavioral immunity in maintaning low PaV1 prevalence in lobsters with and without the loss of shelters caused by a sponge die-off. The 10-yr simulations depict temporal patterns in the number of juvenile lobsters (mean = black line; scale on right y-axis) and the proportion of lobsters infected with PaV1 (mean = red line; grey line = se; scale on left y-axis) in three separate simulations: (top panel) no behavioral avoidance of PaV1 infected lobsters with sponge die-off in Florida Bay, (middle panel) behavioral avoidance of PaV1 infected lobsters and no sponge die-off, and (bottom panel) behavioral avoidance of PaV1 infected lobsters with sponge die-off in Florida Bay. The olive green bar in the top and bottom panels show the timing of the sponge die-off, which created greater aggregation of lobsters within dens. The overall mean (± 1 se) prevalence of PaV1 and abundance of juvenile lobsters (45–50 mm CL) per 1 km^2^ for each simulation are also provided in each panel.

The model predicted that the sponge die-off would cause lobster aggregations per den to nearly triple and lobster abundance in the region to decline by 53% due to increased predation and emigration; the simulations resulted in a mean of 222 juvenile lobsters/ km^2^ when there was no sponge die-off versus 117 juvenile lobsters/ km^2^ with a sponge die-off (Fig 4). Despite the effect of the sponge die-off on lobster abundance and the increased aggregation of lobsters in the remaining dens, the model predicted that there would be virtually no change in the prevalence of PaV1-infected lobsters whether sponge die-offs occurred (0.03 ± 0.036; mean ± 1 se) or not (0.01 ± 0.010; mean ± 1 se)(Fig 4). These predictions are precisely what we observed in the field before and after the sponge die-off. That is, after the sponge die-off we observed fewer lobsters overall and all were concentrated in the remaining dens (mostly rocky holes and isolated coral heads), but the prevalence of PaV1 in the lobster population was unchanged. However, when we disabled behavioral immunity in the model, so that there was no aversion of infected lobsters by uninfected lobsters, a PaV1 epizootic was predicted within a few months. The mean prevalence of PaV1 in juvenile lobsters increased by an order of magnitude in the simulations to an average of nearly 20%, with peaks in viral prevalence of up to 77%.

### Empirical Time-Series of PaV1 Prevalence in Lobsters

**Fig 5:**
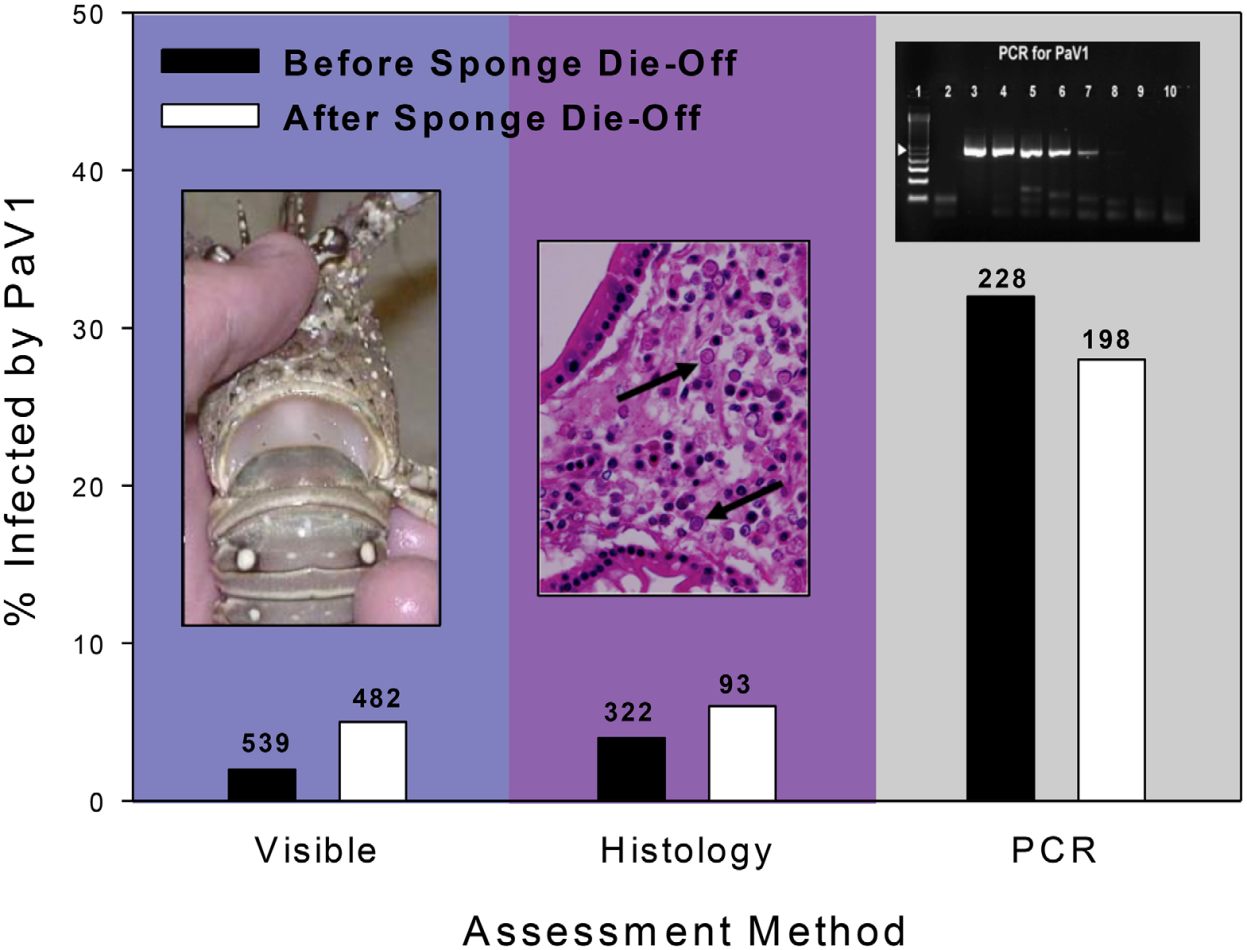
Prevalence of PaV1 in the lobster population before and after the sponge die-off based on three separate measures of infection: visible (i.e., clinical) signs of infection, histological examination of tissue pathology, and PCR analysis of hemolymph. (Photo Insets) Left: juvenile spiny lobster infected by PaV1 showing milky colored hemolymph. Center: stained histological section of hepatopancreatic tissue with arrows pointing to PaV1 infected cells. Right: sensitivity of PaV1 primer set starting at 120 pg DNA in 10-fold dilutions. The number of lobsters tested using each method is shown above each bar in the histogram.

**Fig 6:**
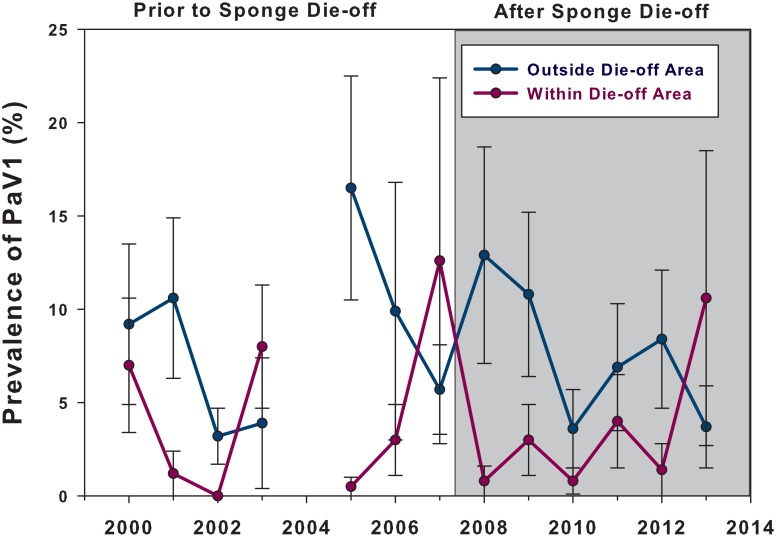
A six year time-series of mean (± 1 se) PaV1 prevalence in juvenile lobster populations at monitoring sites outside (blue line) and within (purple line) the area affected by the sponge die-off. Monitoring was conducted in both areas before the sponge die-off occurred in Sept-Oct 2007 (white background) and after the sponge die-off (grey background). No prevalence data were collected in 2004.

Statistical analysis (separate Chi square goodness of fit tests) revealed that PaV1 prevalence in lobsters was unchanged eight months after the sponge die-off when lobsters were more aggregated in dens as compared to just a few months before (Fig 5) regardless of the method used to quantify PaV1 prevalence (visual inspection: *χ* ^*2*^ = 0.48, df = 1, P = 0.48; histology: *χ* ^*2*^ = 0.53, df = 1, P = 0.48; pcr: *χ* ^*2*^ = 0.61, df = 1, P = 0.46). Annual surveys of PaV1 prevalence at those same 10 sites for another five years following the sponge die-off confirmed that viral prevalence remained relatively stable over time (Fig 6), consistent with our hypothesis and model predictions.

## Discussion

We report results from a large-scale "natural experiment" in which sponges used as dens by juvenile spiny lobsters disappeared after a bloom of cyanobacteria decimated the sponge community across a large portion of the central Florida Keys. In turn, the loss of sponge shelters across the region forced lobsters into unusually large aggregations within the remaining dens, setting in place conditions ripe for the spread of the pathogenic PaV1 virus over the Florida Keys seascape. However, field surveys of PaV1 prevalence in lobsters immediately after the sponge die-off and over the next six years, both within and outside the affected region, showed that PaV1 prevalence instead remained unchanged in the host population. Experimental manipulation of the presence of infected and uninfected lobsters within naturally occurring lobster aggregations demonstrated that even when shelters were scarce, healthy lobsters still vacated dens into which a diseased lobster was introduced, thus preferring an increased risk of predation to an increased risk of infection. Modeling results from an individual-based, spatially-explicit simulation of lobster-PaV1 disease dynamics corroborated the results and mechanisms deduced from our field studies. Specifically, behavioral immunity conferred by the avoidance of infected lobsters by healthy conspecifics appears to have suppressed what otherwise would have been a viral epizootic caused by increased lobster aggregation and viral transmission following the sponge die-off.

PaV1, perhaps in conjunction with similarly influential but unknown pathogens, has been a powerful selective force driving the evolution of flexible social behavior in Caribbean spiny lobsters, including those that confer behavioral immunity against contagious pathogens. The aversion that lobsters display toward infected conspecifics is so strong that they choose to risk predation and search for another den, rather than cohabit a den with an infected conspecific. Indeed, we have previously shown that lobsters will even select a den with the scent of an octopus—a formidable predator whose odor they normally avoid—rather than a shelter with the smell of a diseased lobster [45,46]. The consequence of such dramatic behavior is a population that is remarkably resilient to PaV1 epizootics. Although other ecological factors associated with the sponge die-off could, perhaps, retard the spread of pathogens among lobsters, we can conceive of only a single, viable alternative hypothesis for our findings—predatory culling.

Predatory culling of infected lobsters might minimize the spread of PaV1 among lobsters, and thus contribute to population resilience. We showed in an earlier study [45] that PaV1-infected lobsters are killed twice as often by predators than uninfected lobsters. In host-parasite pathosystems involving intermediate hosts, host behavior is often altered by the parasite to enhance predation risk thus parasite transmission [47–49]. However, in pathosystems with classically defined definitive hosts, like that for the lobster-PaV1 system, predatory culling of infected hosts reduces the spread of pathogens, as demonstrated in studies of captive and wild animal populations [50–52], and supported by theoretical research [53,54]. Predatory culling could explain the steady prevalence of PaV1 among spiny lobsters following the sponge die-off, but only if increased predation on clinically infected (i.e., infectious) lobsters kept density-dependent transmission below some threshold necessary for an epizootic. All of our modeling simulations included differential culling of infected lobsters, but only those that also included behavioral immunity fit our empirical observations of PaV1 prevalence over time.

Disentangling the relative importance of multiple, often interacting factors that contribute to a population's resistance to pathogens is a daunting task, but models that explicitly incorporate complex host-pathogen spatial dynamics offer useful platforms for evaluating alternative, mechanistic hypotheses. The modeling of marine pathogen-host dynamics is still in its infancy, and there is concern that traditional, terrestrial-based epidemiological models may not apply in the sea where host and pathogen life history and modes of pathogen dispersal are fundamentally different [55, 56]. Moreover, few epidemiological models are designed to handle transmission dynamics if complicated by spatio-temporal changes in habitat structure or quality that may alter patterns of disease transmission [11]. Yet, understanding transmission under such circumstances is important for predicting the spread of pathogens in our changing natural environment. The modeling portion of our study offers one such example of the use of a fine-scale, spatially- and behaviorally-explicit epidemiological model to explore host-pathogen dynamics against the backdrop of an altered environmental seascape.

We do not know how common behavioral immunity is in nature, but when present [13, 57] it functions similarly to measures used to control contagious diseases in humans and agroecosystems. In the absence of behavioral immunity, innate or acquired host immunity serves as the crucial line of host defense against infection by pathogens [58]. The immunity of marine hosts, however, is compromised in an increasingly warm, eutrophic, and acidic sea where existing pathogens may flourish and new diseases emerge [59, 60]. Marine pathogens tend to spread rapidly at rates of up to 3000–10,000 km/yr, eclipsing many of the fastest spreading terrestrial pathogens [61]. Thus, the potential for widespread epizootics is of special concern in the sea. In the early 1980’s, for example, an unknown pathogen killed over 90% of the long-spined sea urchins throughout the Caribbean and contributed to a widespread ecological phase-shift from coral- to algae-dominated coral reefs [62]. That dramatic series of events, along with similarly destructive disease-related collapses of coral and fish communities have placed marine diseases on center stage. Once a curiosity among marine scientists, diseases are now recognized as a major ecological and evolutionary force in the sea. Our study demonstrates that for at least some species, behavioral immunity can help check the spread of disease through the quarantine of infected individuals, and thus serves as a behavioral “skirmish line” against pathogens by preventing their invasion of hosts.
